# Incidental Finding of a Persistent Left Superior Vena Cava During Permanent Dual-Chamber Pacemaker Implantation: A Case Report

**DOI:** 10.7759/cureus.72865

**Published:** 2024-11-01

**Authors:** Shehnoor Kaur, Shaik Firdaus, Jhiamluka Solano, Sachin Manjunath, Adnan Ahmed

**Affiliations:** 1 Emergency Medicine, Scunthorpe General Hospital, Scunthorpe, GBR; 2 Council, Academy of Medical Educators, Cardiff, GBR; 3 Cardiology, Scunthorpe General Hospital, Scunthorpe, GBR; 4 Cardiology, Hull University Hospital (Castle Hill Hospital), Hull, GBR

**Keywords:** atrial fibrilation, dual chamber pacemakers, heart failure with preserved ejection fraction (hfpef), high-degree av block, persistent left superior venacava

## Abstract

Persistent left superior vena cava (PLSVC) is a rare congenital venous anomaly. It is often asymptomatic and has atypical venous drainage that can complicate central venous catheterisation, pacemaker implantation, and cardiac surgeries. In most cases, the PLSVC drains into the right atrium via the coronary sinus, but in a minority of cases, it drains into the left atrium, leading to a right-to-left shunt, which can cause mild hypoxia or paradoxical embolism. Due to its abnormal anatomy, PLSVC can complicate lead placement during permanent pacemaker (PPM) insertion. Lead navigation becomes more complex, sometimes necessitating alternative lead placement techniques or imaging guidance to ensure proper functionality. In most cases, the PLSVC is identified incidentally during the initial venogram.

We present the case of a 64-year-old male with sarcoidosis, hypercholesterolemia, hypertension, hepatitis, and recent atrial fibrillation (AF) who presented for elective direct current cardioversion (DCCV). Pre-DCCV, the ECG showed AF with a slow ventricular response, and following a 200-joule synchronised shock as per local protocol, sinus rhythm was restored. Post-DCCV ECG showed a first-degree AV block, which progressed to an intermittent 2:1 block, leading to a decision to implant a dual-chamber PPM. An echocardiogram revealed normal left ventricular function, a dilated left atrium and normal right ventricle, mild tricuspid regurgitation, and a possible patent foramen ovale (PFO). A venogram performed during PPM implantation revealed a PLSVC, which posed challenges in lead placement. Despite initial success, a post-procedure chest X-ray revealed displacement of the atrial lead, prompting a successful repositioning. The patient remained stable and asymptomatic; outpatient follow-ups showed satisfactory PPM function.

PLSVC is a congenital anomaly arising from incomplete regression of the left anterior cardinal vein during embryonic development. Though it is often discovered incidentally, the anomaly becomes clinically significant during procedures such as pacemaker implantation due to its impact on venous anatomy and lead placement. This case also underscores the need for specialised techniques when managing patients with PLSVC during device implantation. Given the abnormal venous pathway, alternative strategies such as utilising the coronary sinus or imaging guidance, like fluoroscopy, may be necessary to ensure proper lead placement and avoid complications such as lead displacement or venous thrombosis. The literature supports using advanced imaging modalities and tailored surgical approaches to improve outcomes in patients with PLSVC. Ultimately, this case illustrates the complexity of cardiac device implantation in the presence of venous anomalies and highlights the importance of individualised procedural planning to optimise patient care and reduce the risk of complications.

## Introduction

Persistent Left Superior Vena Cava (PLSVC) is a venous abnormality found in approximately 0.3-0.5% of the general population and 4.5% of individuals with congenital heart defects. Although PLSVC is often asymptomatic and discovered incidentally, it can present significant challenges during specific medical procedures [1, 2]. Its atypical venous drainage can complicate central venous catheterisation, pacemaker implantation, and cardiac surgeries, as the altered anatomy may hinder access to the right atrium and pulmonary circulation. In 92% of the cases, PLSVC drains into the right atrium via the coronary sinus and in about 8%, it drains into the left atrium, potentially causing a right-to-left shunt, which could result in mild hypoxia or paradoxical embolism [3].

During permanent pacemaker (PPM) insertion, PLSVC can complicate lead placement due to its abnormal anatomy [2]. Since the left SVC drains into the coronary sinus instead of directly into the right atrium, lead navigation can become more complex, often requiring adjustments in technique [4]. In some instances, alternative lead placement methods or imaging guidance may be necessary to ensure proper positioning and functionality [4]. Recognising PLSVC before such procedures is essential for clinicians to plan appropriately, minimise procedural risks, and achieve successful outcomes for the patient [3].

## Case presentation

**Figure 1:**
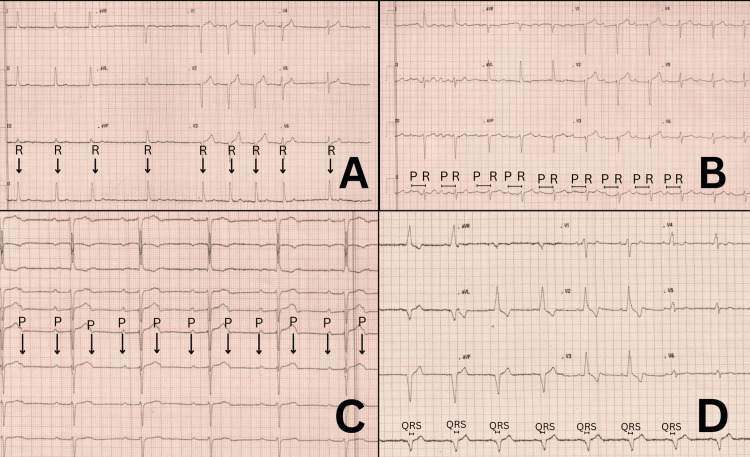
ECG series during admission. A. ECG showing atrial fibrillation with a slow ventricular response evidenced in the lead II by the irregular R wave distance. B. Post direct current cardioversion (DCCV) ECG showing sinus rhythm with first-degree atrioventricular (AV) block evidenced in the lead II by the prolonged PR interval segment (>200ms); C. ECG showing intermittent 2:1 AV block evidenced in the lead V3 by the non-conducted P wave and absence of a QRS complex and superimposed P wave in the T waves; D. Post dual-chamber implant ECG as evidenced in the lead II by a wide QRS complex.

A 64-year-old male with a background of sarcoidosis with Heerfordt's syndrome, hypercholesterolemia, hypertension, hepatitis, and a recent diagnosis of atrial fibrillation (AF) was scheduled for elective direct current cardioversion (DCCV). Initial ECG revealed AF with a slow ventricular response (SVR) (Figure 1A). The rest of the investigations were normal. A synchronised 200-joule shock successfully terminated the arrhythmia. However, the post-cardioversion ECG indicated a first-degree atrioventricular (AV) block (Figure 1B). He was subsequently admitted for cardiac monitoring and observation.

**Figure 2:**
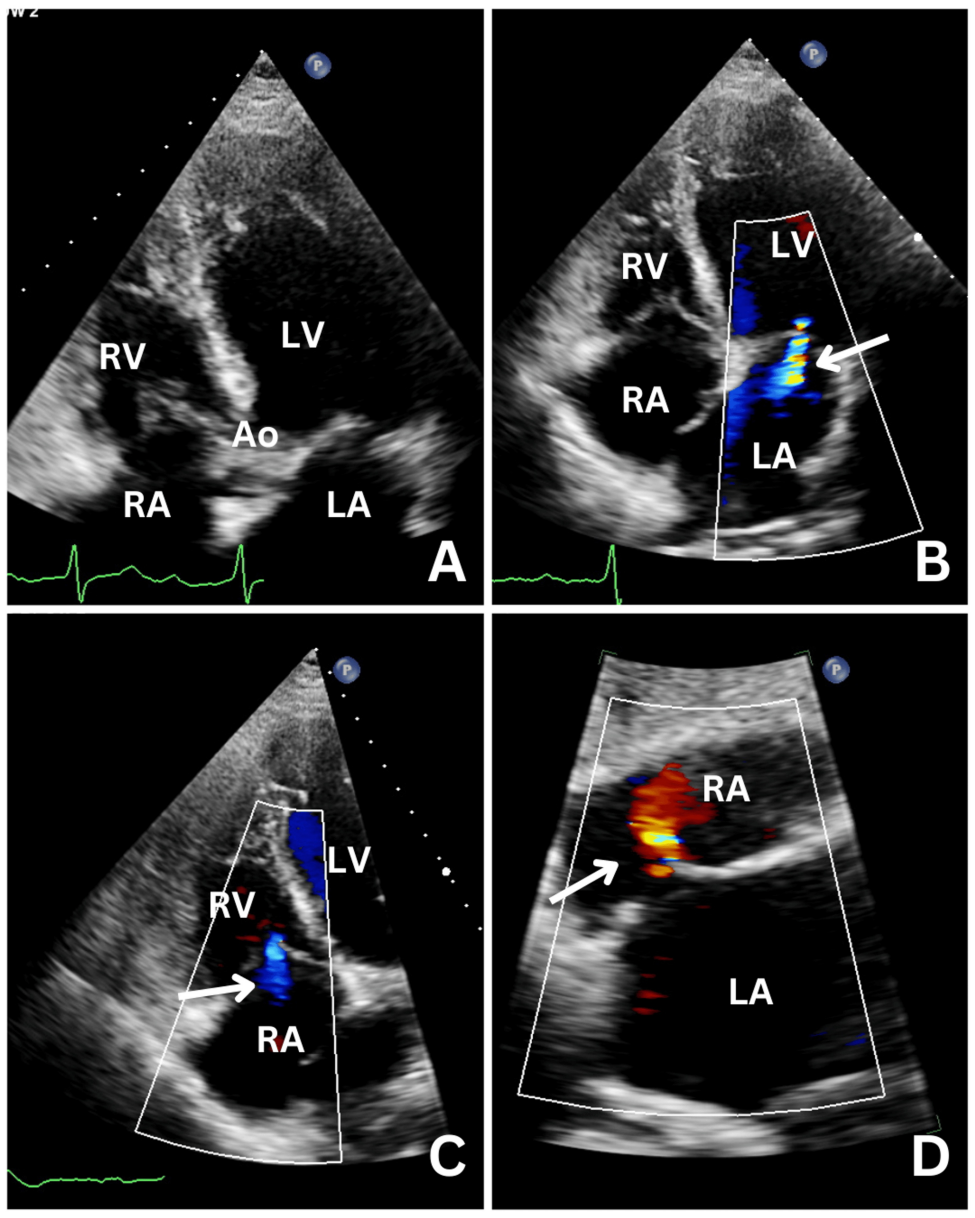
Transthoracic Echocardiography A. Apical four-chamber view shows a dilated Right Ventricle (RV), B. Arrow shows functional mitral regurgitation and dilated atriums in apical four-chamber view, C. Arrow shows mild tricuspid regurgitation and dilated right atrium in apical four-chamber view, and D. Arrow shows a small jet of colour crossing the interatrial septum suggesting the presence of patent foramen ovale (PFO) in subcostal view. LV: left ventricle; LA: left atrium; RA: right atrium; Ao: aorta

During admission, blood tests revealed a C-reactive protein (CRP) of 0.5 mg/L, normal liver function tests (LFTs), an estimated glomerular filtration rate (eGFR) of 82 mL/min, and an glycated haemoglobin (HbA1C) of 3%. An echocardiogram performed post-cardioversion showed a normal left ventricular ejection fraction (LVEF) of 60%, with a dilated left atrium (LA) and normal size and function of the right ventricle (RV). Systolic function was preserved, and mild tricuspid regurgitation (TR) was present. The echocardiogram also raised the possibility of a patent foramen ovale (PFO) (Figure 2). Post cardioversion, the patient remained hemodynamically stable with a heart rate of 54 bpm and a blood pressure of 130/75 mmHg. During his stay, the first-degree AV block evolved into a high-degree AV block (AVB), identified as an intermittent 2:1 AV block (Figure 1C). Due to the non-resolving high-degree AVB, a decision was made to implant a dual-chamber permanent pacemaker (PPM).

**Figure 3:**
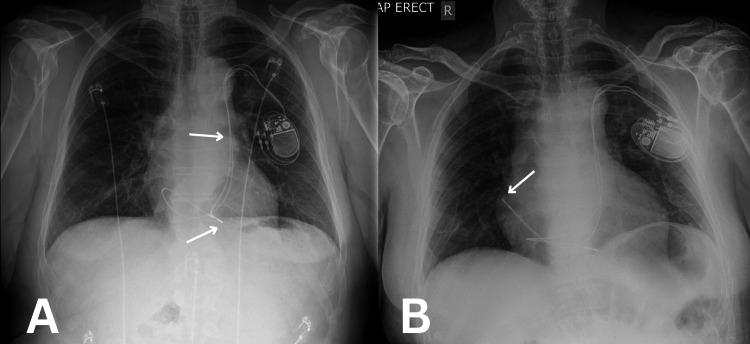
Post-procedure chest X-rays. A. Post permanent pacemaker (PPM) implant anteroposterior (AP) chest X-ray shows evidence of left persistent superior vena cava (LPSVC) due to the trajectory of the PPM leading down to the right atrium and ventricle (top arrow). The bottom arrow points to the displaced right atrium lead. B. Post-lead repositioning AP chest X-ray shows a successful right atrium lead positioning (arrow).

The initial venogram during the PPM implant revealed a persistent left superior vena cava (LSVC) and no evident left brachiocephalic vein (Video 1). PPM implantation was successful with adequate stability checks. Following pacemaker implantation, the patient remained stable, reporting no shortness of breath, dizziness, or pedal oedema. He could mobilise without difficulty, and the pacemaker insertion site was clean and non-tender, with no signs of infection. A pacemaker check confirmed normal functioning. However, a post-implantation chest X-ray revealed the displacement of the atrial lead (Figure 3A). Despite these findings, the patient remained asymptomatic and stable. The displaced atrial lead was successfully repositioned (Figure 3B). Post lead repositioning, the PPM implant site developed a haematoma, which resolved. The patient was followed as an outpatient, and PPM checks remain satisfactory.

**Video 1:** Pre pacemaker implant right venogram showing the persistent left superior vena cava.

## Discussion

The presence of a persistent left superior vena cava (LSVC) is a rare congenital anomaly that occurs due to an abnormality in the embryonic development of the venous system [3]. Typically, during embryogenesis, the left cardinal vein regresses, leaving the right superior vena cava (SVC) as the dominant vessel, returning blood from the upper body to the heart [3]. In cases of persistent LSVC, the left cardinal vein fails to regress and remains as an additional or dominant venous return pathway [2]. The anomaly occurs in about 0.3-0.5% of the general population, though it is more common in patients with congenital heart defects, with prevalence rates as high as 4.5% in this population [1]. While a persistent LSVC typically drains into the right atrium via the coronary sinus, in some cases, it may connect directly to the left atrium, leading to a right-to-left shunt, which can be clinically significant [3].

Clinically, a persistent LSVC is often asymptomatic and discovered incidentally during imaging or surgical procedures [3]. However, it can present with symptoms when associated with other congenital cardiac anomalies, such as atrial septal defects or patent foramen ovale (PFO), which may allow for paradoxical embolism [5]. In this case study, the patient was noted to have both a dilated coronary sinus and a possible PFO on echocardiography. The clinical relevance of a persistent LSVC becomes significant during invasive cardiac procedures, especially pacemaker or defibrillator lead implantation [4]. The abnormal venous anatomy can complicate the placement of pacemaker leads, as was seen in this patient, where atrial lead displacement occurred post-implantation [6].

Regarding permanent pacemaker (PPM) insertion, the persistent LSVC poses technical challenges [4]. Standard pacemaker lead insertion is done through the right SVC into the right atrium, but in patients with a persistent LSVC, the leads must travel through an abnormal venous pathway, which may affect the stability and positioning of the leads [7]. In this case, the lead displacement was discovered on the post-implantation chest X-ray, prompting lead repositioning. The literature describes various adaptations for PPM placement in the setting of persistent LSVC to prevent complications, including using the coronary sinus or adjusting the lead length and positioning to accommodate the altered anatomy [8].

**Figure 4:**
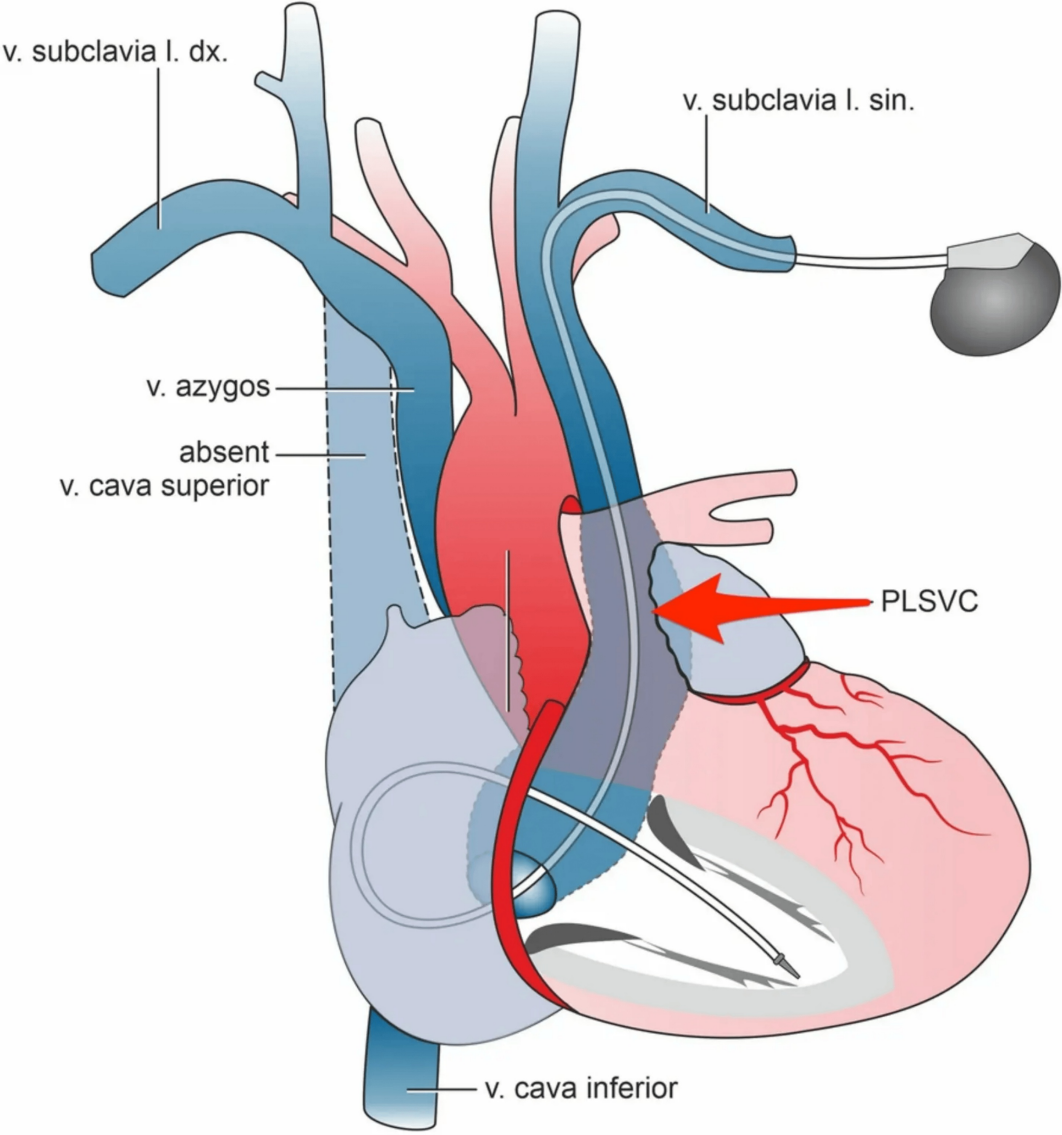
Illustration of final right ventricular lead position. The right ventricular lead trajectory is illustrated through the persistent left superior vena cava (PLSVC). v. = vena; l. dx. = lateris dextri; l. sin = lateris sinistri. Source: Plášek et al. [9]. Available under Creative Commons Attribution 4.0 International License (https://creativecommons.org/licenses/by/4.0/deed.en).

Among the different techniques for PPM implantation in patients with a persistent LSVC, using the coronary sinus as the primary route is often recommended due to its proximity to the right atrium (Figure 4) [9]. A standard transvenous approach was initially used in this case, but lead displacement occurred, likely due to the altered anatomy. Other recommended techniques include guiding the lead using fluoroscopy or contrast-enhanced imaging to visualise venous pathways better or performing epicardial lead placement in more complex cases. In the literature, success with fluoroscopic guidance and coronary sinus cannulation has been well-documented, but each approach must be individualised based on the patient's anatomy [10]. 

Complications associated with pacemaker insertion in the presence of a persistent LSVC are more common than in normal anatomy [11]. These include lead displacement, as seen in this patient, as well as an increased risk of arrhythmias, coronary sinus perforation, and venous thrombosis due to altered venous flow [1]. While lead displacement is often correctable with repositioning, the risk of recurrent displacements is higher [12]. A detailed preoperative assessment and advanced imaging modalities are essential to distinguish the venous anatomy and can help mitigate these risks, but LSVC remains a significant procedural challenge [4,12].

## Conclusions

This case highlights the significance of identifying and managing a persistent left superior vena cava (PLSVC) in patients undergoing invasive cardiac procedures, such as pacemaker implantation. In this 64-year-old patient, the presence of a PLSVC was incidentally discovered after a permanent pacemaker (PPM) insertion for an atrioventricular block, during which lead displacement was noted. Despite the patient being asymptomatic and stable, the PLSVC presented a challenge regarding lead stability and positioning, illustrating the importance of recognising this venous anomaly to avoid procedural complications. The patient's echocardiogram findings, which showed a possible patent foramen ovale (PFO), further emphasised the need for thorough pre-procedural evaluation in patients with such anatomical variants, as these can have implications on both the procedure and postoperative outcomes. 

This case also underscores the need for specialised techniques when managing patients with PLSVC during device implantation. Given the abnormal venous pathway, alternative strategies such as utilising the coronary sinus or imaging guidance, like fluoroscopy, may be necessary to ensure proper lead placement and avoid complications such as lead displacement or venous thrombosis. The literature supports using advanced imaging modalities and tailored surgical approaches to improve outcomes in patients with PLSVC. Ultimately, this case illustrates the complexity of cardiac device implantation in the presence of venous anomalies and highlights the importance of individualised procedural planning to optimise patient care and reduce the risk of complications.
